# miR-10a Regulates Proliferation of Human Cardiomyocyte Progenitor Cells by Targeting GATA6

**DOI:** 10.1371/journal.pone.0103097

**Published:** 2014-07-28

**Authors:** Dandan Liang, Lixiao Zhen, Tianyou Yuan, Jian Huang, Fangfei Deng, Wuyahan, Hong Zhang, Lei Pan, Yi Liu, Erlinda The, Zuoren Yu, Weidong Zhu, Yuzhen Zhang, Li Li, Luying Peng, Jun Li, Yi-Han Chen

**Affiliations:** 1 Key Laboratory of Arrhythmia of the Ministry of Education of China, East Hospital, Tongji University School of Medicine, Shanghai, China; 2 Institute of Medical Genetics, Tongji University, Shanghai, China; 3 Department of Cardiology, East Hospital, Tongji University, Shanghai, China; 4 Department of Pathology and Pathophysiology, Tongji University School of Medicine, Shanghai, China; Duke University, United States of America

## Abstract

microRNAs (miRNAs) play essential roles in cardiogenesis. The altered expression of miRNAs can result in cardiac malformations by inducing abnormalities in the behavior of cardiac cells. However, the role of miR-10a in the regulation of cardiomyocyte progenitor cells (CMPCs) remains undetermined. In the present study, we found that up- or down-regulation of miR-10a inhibited or promoted the proliferation of human CMPCs, respectively, without affecting their differentiation toward cardiomyocytes. miR-10a bound to GATA6 directly and reduced GATA6 expression. Over-expression of GATA6 greatly attenuated the miR-10a-mediated inhibitory effect on the proliferation of human CMPCs. Thus, our results indicate that miR-10a could effectively modulate the proliferation of human CMPCs by targeting GATA6. The finding provides novel insights into the potency of miR-10a during heart development.

## Introduction

Cardiac progenitor cells that originate from the mesoderm play essential roles in heart morphogenesis. Their behaviors are finely orchestrated in every step of cardiac development [1]. In the initiative phase of cardiogenesis, the progenitor cells actively proliferate and are recruited to form a progenitor pool, which contributes to the development of the primary heart tube [2]. At the stage of looping morphogenesis, cardiac progenitor cells migrate toward both poles of the heart tube and promote its rapid growth [3], [4]. Subsequently, cardiomyocytes derived from cardiac progenitor cells begin to enlarge and proliferate to ensure the ballooning mode formation of the cardiac chambers [2], [5]. In the stage of ventricular development, cardiomyocytes further specify to form the trabecular and compact layer of the ventricles [6]. Finally, progenitor cell-based extension of the outflow tract (OFT) is critical for separation and correct alignment of the great vessels [7]. Obviously, dysregulation of the progenitor cell behaviors can cause cardiac malformations ranging from minor morphologic defects to a complete loss of the cardiac structures derived from them [7], [8].

miRNAs are a class of small non-coding RNAs that target the 3′UTR of mRNAs in a sequence-specific manner. They have been shown to participate in the regulation of cardiogenesis [9]. Disruption of the Dicer allele, which is essential for miRNAs processing, in early stage of cardiogenesis results in lethal cardiac defects, indicating the requirement of miRNAs in cardiac development [10]. Nevertheless, the role of individual miRNAs in modulating cardiac progenitor cell behavior is not fully understood. miR-10a has been suggested to regulate retinoid acid-induced smooth muscle cell (SMC) differentiation from ES cell and endothelial progenitor cell senescence [11], [12]. It also contributes to the plasticity of helper T cells [13] and the proliferation, differentiation and migration of cancer cells [14], [15]. However, the role of miR-10a in cardiac progenitor has not been elucidated.

In the present study, we attempted to investigate whether miR-10a regulates cardiac progenitor cell behavior, and if so, the potential mechanism of the regulation. Using human cardiomyocyte progenitor cells (hCMPCs), we show that miR-10a can regulate the proliferation of hCMPCs by targeting GATA6, and it does not influence the differentiation of hCMPCs toward cardiomyocytes.

## Materials and Methods

This study was approved by the ethical committees of East Hospital, Tongji University School of Medicine (Protocol Number: 2012-DF-30). Using standard informed consent procedures, the written informed consents from the mothers were obtained. The experiments were carried out in accordance with the Declaration of Helsinki (2008) of the World Medical Association.

### hCMPC Isolation and Culture

hCMPCs were acquired and characterized as our previous study [16]. Briefly, the isolated cells from human fetal heart tissue were sorted by flow cytometry using a mouse anti-Sca-1 antibody (eBioscience, USA) and then cultured in growth medium (GM), which was changed every other day, and splitted as soon as 70–90% confluence was achieved [16], [17].

### hCMPC Transfection

hsa-miR-10a mimics (5′-UACCCUGUAGAUCCGAAUUUGUG-3′ and 5′-CAAAUUCGGAUCUACAGGGUAUU-3′), inhibitor (5′-CACAAAUUCGGAUCUACAGGGUA-3′) and negative scramble (mock) were purchased from Genepharm. These oligonucleotides were transfected into hCMPCs using siPORT NeoFX transfection agent (AM4511, Ambion, USA) following the manufacturer's instructions. Up/downregulation of miR-10a was confirmed by qRT-PCR after 48 h of transfection.

### Cytotoxicity and Proliferation

Cytotoxicity was assessed with the Cell Counting Kit-8 (CCK-8, Dojin, Japan). hCMPCs were transfected for 48 h, and 10 µl of the CCK-8 solution was added to each well of the plate. After incubation for 2 h at 37°C, the absorbance was measured at 450 nm using the Spectra Max5 (Molecular Devices, USA).

Cell Proliferation ELISA combined with BrdU (Colorimetric) (11647229001, Roche, Switzerland) were used to evaluate hCMPC proliferation. hCMPCs were separately transfected with miR-10a mimics (50 nM) or inhibitor (50 nM) for 48 h. BrdU was added to the growth medium, and the mixture was incubated overnight. After removing the growth medium, cells were fixed and denatured using FixDenat for 30 min. Then, anti-BrdU-POD that binds the BrdU incorporated in the newly synthesized DNA was added. Finally, substrate was introduced, and the absorbance at 370 nm and 492 nm (reference) was detected with the Spectra Max5 (Molecular Devices, USA). The proliferation rate was further verified using the EdU assay (C10339, Invitrogen, USA). The cell counting experiment was also performed to measure hCMPCs proliferation with crystal violet staining (Biotime, China).

### Measurement of hCMPC Cell Cycle

hCMPCs were transfected for 48 h and then trypsinized with 0.25% trypsin (non-EDTA) and fixed with pre-chilled 70% ethanol at −20°C overnight. Before measuring the cell cycle using flow cytometry, the cells were washed with PBS and incubated with 50 µg/mL propidium iodide (P4170, Sigma-Aldrich, USA) in PBS at 37°C for 30 min. Data were analyzed with Multicycle version 6.0. The expression of cell cycle-related genes were also detected by RT-PCR.

### Induced Differentiation of hCMPCs

hCMPCs were induced to differentiate into cardiomyocytes according to the protocol described previously [17]. First, 5 mM 5-azacytidine (A2385, Sigma, USA) was added for three days; then, hCMPCs were treated with transforming growth factor (TGF) -β1 (1 ng/ml, 100-21c, PeproTech, USA) and ascorbic acid (100 µM, A5960, Sigma, USA). After treatment for 14 days, RNA was extracted for RT-PCR. We detected the expression of transcription factors indicative of cardiomyocyte predisposition (MEF2C, GATA-4, Nkx-2.5) and genes encoding sarcomere assembly proteins (α-Actinin, β-MHC). In addition, sarcomeric proteins α-Actin (A7811, Sigma, USA) and Troponin I (ab52862, Abcam, USA) were stained to quantify the degree of differentiation based on a previously provided protocol [17]. The cells were photographed with the Live Cell Imaging System (Leica AF 7000, Leica).

### Western Blotting

hCMPCs were transfected with miR-10a mimics or inhibitor for 48 h. Then, the cells were harvested and lysed with RIPA buffer (Biotime, China) for protein extraction. Proteinase inhibitors were used to minimize protein decomposition. Western blotting was performed using NuPAGE 4%–12% Bis-Tris Gel (NP0335BOX, Invitrogen, USA). The blots were incubated with primary antibodies anti-GATA6 (5851, Cell Signal Technology, USA), anti-caspase 3 (Cell Signaling, USA) and anti-GAPDH (Cell Signaling, USA) at 4°C overnight to detect the relative protein expression levels, and a fluorescent secondary antibody (072-07-15-06, KPL, USA) was applied to label the primary antibody. The labeled bands were visualized with Oddesey (Li-Cor).

### miR-10a Target Gene Analysis

Potential targets of human miR-10a were searched using Targetscan (http://mirtarbase.mbc.nctu.edu.), an online target prediction database. Then, the luciferase assay and western blotting were used to verify the target. A luciferase expression vector containing a predicted binding site of miR-10a in human GATA-6 3′UTR (5′-ACAGGGTA-3′) was cotransfected with mock or miR-10a mimics into 293 cells using Lipofectamine 2000 (11668-019, Invitrogen, USA). Then, 48 h after transfection, the luciferase assay was performed using a dual luciferase reporter assay system (E2920, Promega, USA). The same plasmid, except that it was devoid of the predicted binding site (5′-ACAGGGTA-3′), was cotransfected with miR-10a mimics to verify the specificity of the binding. To further validate whether miR-10a influences the abundance of GATA6 expression, hsa-miR-10a mimics, inhibitor or mock were transfected into hCMPCs; 48 h later, the cells were harvested for protein detection.

### Modulation of GATA6 in hCMPCs

To construct the GATA6 overexpression plasmid, the entire CDS region of GATA6 (NCBI reference sequence: NM_005257.4) was subcloned into the PCDNA 3.0 vector. The GATA6 overexpression plasmid was transfected into hCMPCs using Lipofectamine 2000 (11668-019, Invitrogen, USA), which is also used for the co-transfection experiments. GATA6 siRNA (5′-CCUCUUCUAACUCAGAUGATT-3′ and 5′-UCAUCUGAGUUAGAAGACGTT-3′) was purchased from GenePharma. Protein expression of GATA6 was measured by western blot after different treatment. Then, a BrdU and EdU assay was performed to observe how GATA6 influences the proliferation of hCMPCs.

### miR-10a expression pattern determination

To determine the expression pattern of miR-10a, Embryo day 9.5 (E9.5), E11.5, E13.5, E15.5, E17.5, E19.5 and Postnatal day 0 (P0) mouse hearts were harvested for total RNA extraction using the mirVana miRNA Isolation Kit (AM1561, Ambion, USA). RNA was then reverse transcribed with the RT Kit (218160, QIAGEN, Germany), and miR-10a was quantified using the miScript SYBR Green PCR kit (218073 QIAGEN, Germany) with the primer purchased from QIAGEN (MS00032242, Germany).

### Statistical Analysis

Data were expressed as the means ± S.E. An independent-samples t-test, chi-square test or one-way ANOVA were used to assess the differences among the experimental groups. If a significant difference was observed, Bonferroni's post-hoc test was performed to identify the groups with significant differences. P-values less than 0.05 were considered to be statistically significant.

## Results

### miR-10a Regulates the Proliferation of hCMPC

To investigate the potential effects of miR-10a on hCMPC, we first used different concentrations of miR-10a mimics and inhibitor to determine the optimal transfection concentration and to avoid cytotoxic effects. The CCK-8 test showed that 30 nM and 50 nM of either oligonucleotide do not affect the metabolic activity of hCMPCs, whereas 100 nM significantly decreased the cells metabolic activity, suggesting the concentration is cytotoxic (**Figure S1 in File S1**). Therefore, the concentration of 50 nM seems to be optimal for our study. Next, we verified that 50 nM mimics or inhibitor could successfully up- or downregulate miR-10a in hCMPCs (**Figure S2 in File S1**).

**Figure 1:**
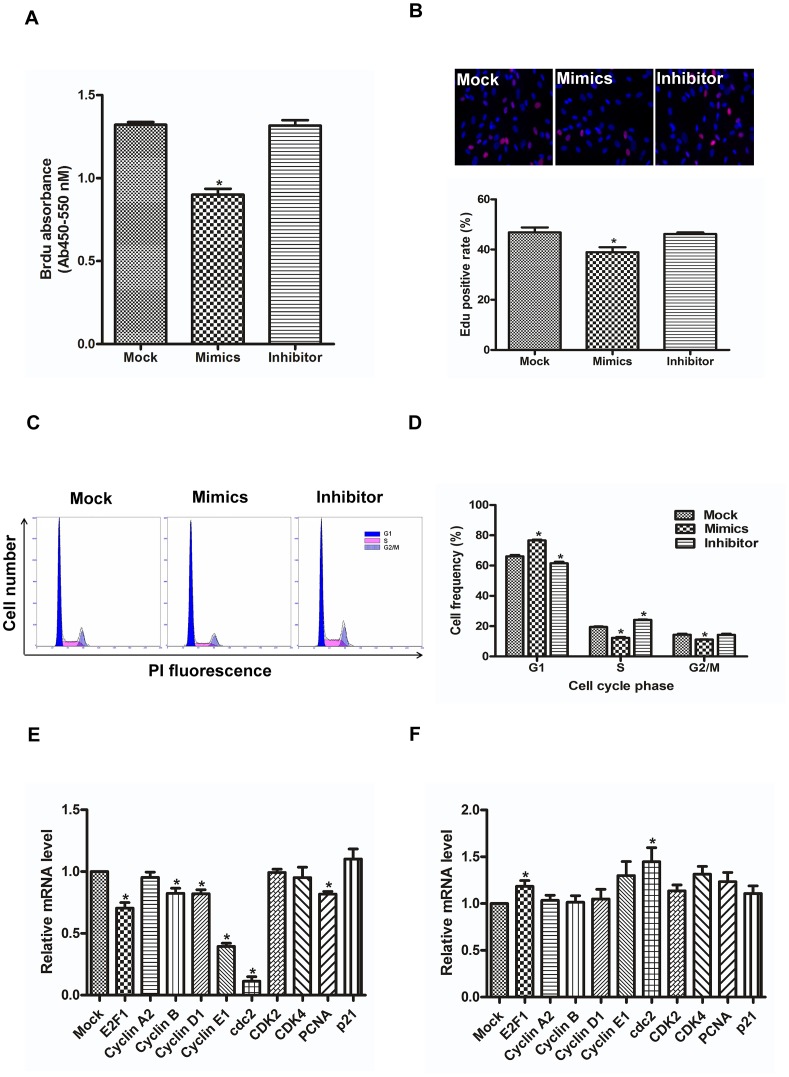
miR-10a reduces proliferation of hCMPCs. A. miR-10a mimics decrease the BrdU incorporation rate of hCMPCs, whereas a miR-10a inhibitor does not significantly affect the process. *P<0.05, n = 6. B. miR-10a inhibits EdU incorporation of hCMPCs. Representative image of hCMPCs transfected with mock, miR-10a mimics or inhibitor stained with DAPI (blue) and EdU (red) (×200). *P<0.05, n = 5. C. Representative flow cytometry results of hCMPC manipulated with mock, miR-10a mimics or miR-10a inhibitor. hCMPCs overexpressing miR-10a show G1/S blocking, and the inhibition of miR-10a promotes G1/S transition compare with mock. D. Data collected from C. *P<0.05, n = 3. E. Relative expression of cell cycle regulatory genes in hCMPCs transfected with miR-10a mimics. F. The same set of genes was measured in hCMPCs with inhibited miR-10a. The expression level of GAPDH was used as the control. *P<0.05, n = 5.

Proliferation of cardiac progenitor cells is emerging as a critical checkpoint in the regulation of cardiac development [18]. We manipulated the expression of miR-10a in hCMPCs, and observed its effect on proliferation. The BrdU ELISA assay indicated that miR-10a decreases the BrdU incorporation rate by 31.82% compared with mock, but the miR-10a inhibitor does not interrupt this process (**Figure 1A**). Based on another set of experiments using the EdU test, miR-10a decreases the EdU incorporation rate by 16.76% (**Figure 1B**), whereas the inhibitor does not produce a significant difference from control. The results in cell counting experiment were consistent with that by Brdu and EDU staining (**Figure S3 in File S1**). Next, whether miR-10a inhibits cell cycle progression of hCMPCs was investigated. As shown in **Figure 1C and 1D**, hCMPCs overexpressing miR-10a display 15.81% higher proportion of G0/G1 phase but 36.85% and 22.76% lower proportion of S and G2/M phase, respectively compared with mock, suggesting that miR-10a inhibits G1/S transition. To confirm this finding, we then examined the expression of specific cell cycle regulators. We found that miR-10a mimics significantly downregulates E2F-1, cyclin B, cyclin D1, cyclin E1 as well as Cdc2 and PCNA but does not significantly influence the expression of P21 (**Figure 1E**). On the other hand, inhibition of miR-10a causes upregulation of E2F-1, Cdc2, CDK2, CDK4 and PCNA (**Figure 1F**). Together, miR-10a inhibits the proliferation of hCMPC by decreasing its cell cycle progression. Futhermore, we detected the protein expression of caspase 3 in the hCMPCs and found miR-10a mimics did not affect the protein levels (**Figure S4 in File S1**). This evidence suggests that the decreased hCMPCs proliferation by miR-10a is not involved in cell apoptosis.

**Figure 2:**
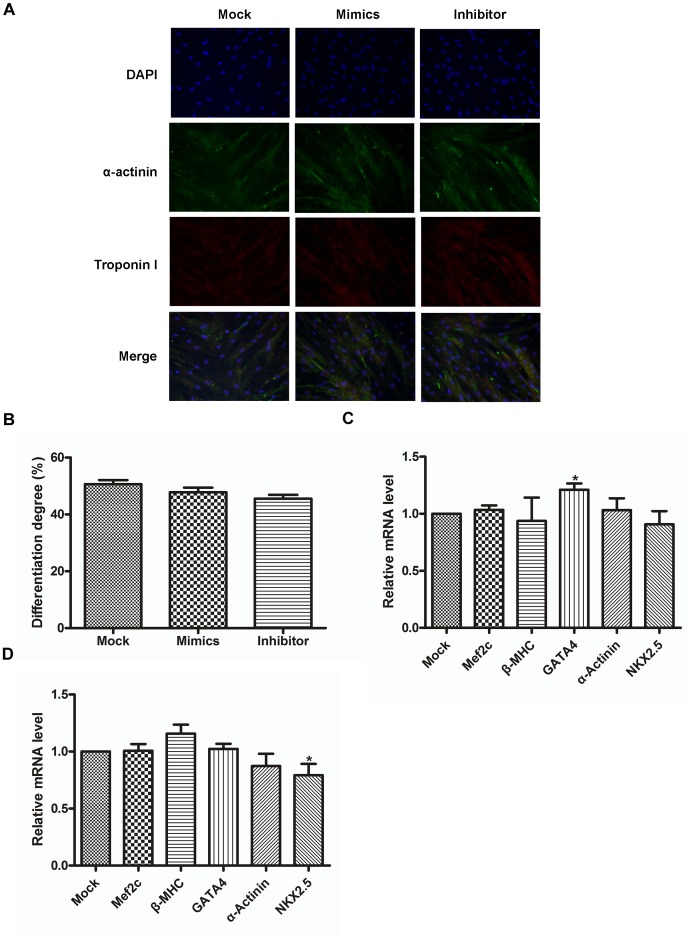
miR-10a does not influence hCMPC differentiation toward cardiomyocytes. A. Representative image of differentiating hCMPCs with manipulated expression of miR-10a or mock. Cells were stained with DAPI, α-actinin and Trop I (×200). B. Data collected from A. *P<0.05, n = 6. C. Relative expression of cardiomyocyte markers in differentiating hCMPCs transfected with miR-10a mimics. D. The same markers were tested in hCMPCs with inhibited miR-10a. The expression level of GAPDH was used as the control. *P<0.05, n = 6.

The inhibition of cell expansion is the prerequisite for differentiation of progenitor cells [1]. Therefore, we analyzed the potential effect of miR-10a on the differentiation of hCMPCs into cardiomyocytes by quantifying lineage-specific markers. We used the expression of sarcomeric proteins, troponin-I and α-actinin, as markers for differentiation, and the results revealed that miR-10a does not significantly change the ratio of differentiating cells (**Figure 2A, B**). We did not detect differences in the transcription levels of other cardiomyocyte markers such as MEF2C, GATA-4, α-actinin, β-MHC and NKX-2.5 (**Figure 2C, D**). Collectively, these findings suggest that miR-10a reduces hCMPC proliferation but does not influence their differentiation into cardiomyocytes.

### miR-10a Binds to GATA6 and Decreases its Expression

**Figure 3:**
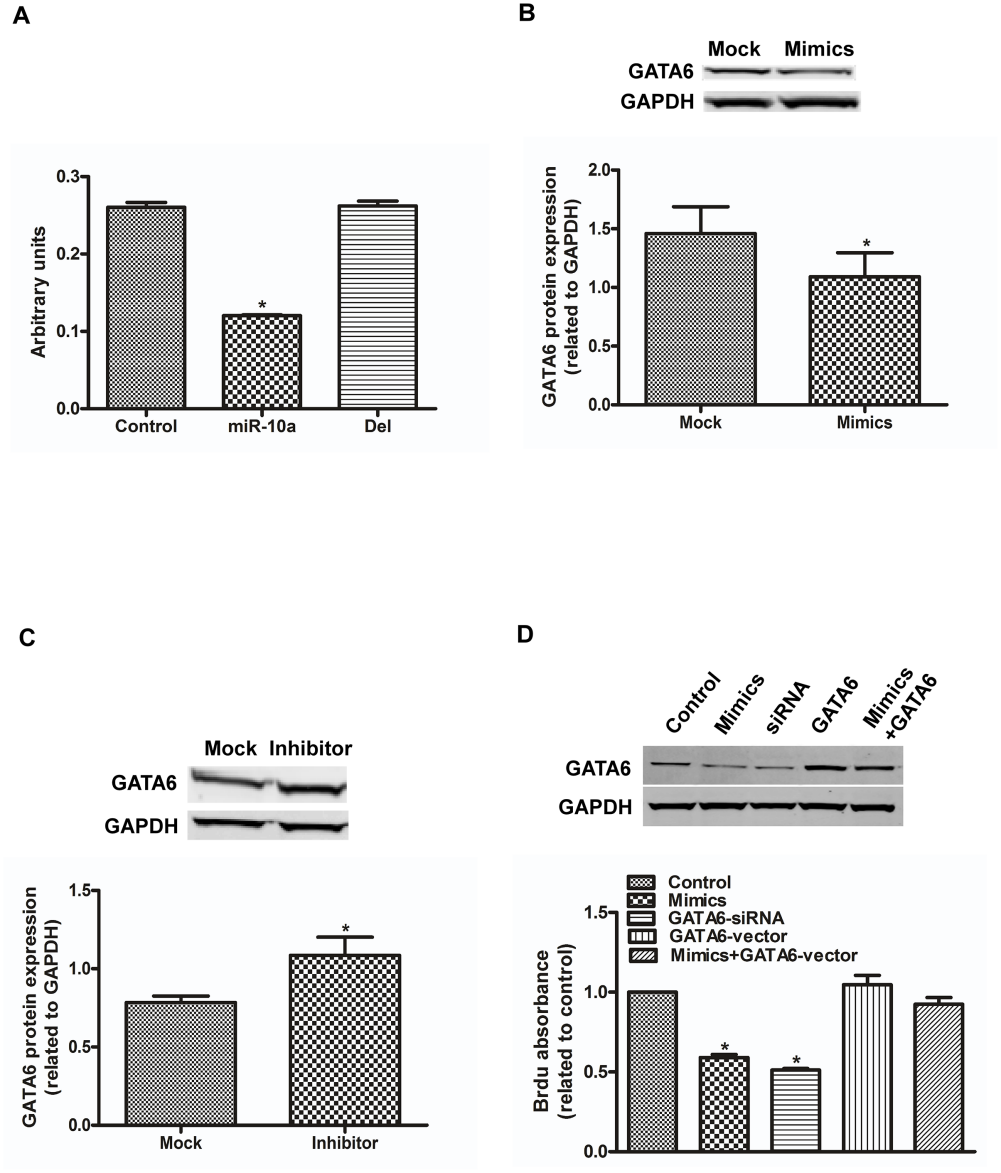
miR-10a binds to GATA6 and suppresses GATA6 expression. A. Pmir- glo dual luciferase plasmid containing a wild type or mutant GATA6-3′UTR was cotransfected with miR-10a mimics or mock into 293 cells. The relative luciferase activity was shown. *P<0.05, n = 5. B. Western blot shows the expression of GATA6 in hCMPCs transfected with mock and miR-10a mimics. C. The same experiment with mock and inhibitor. Quantification of protein expression was normalized to GAPDH. *P<0.05, n = 3. D. Overexpression of GATA6 rescues the miR-10a inhibitory effect on hCMPC proliferation. Protein expression of GATA6 with different treatment was showed. BrdU absorbance of hCMPCs transfected with mock, miR-10a mimics and siRNA that downregulated GATA6 are shown. Similar to miR-10a, the downregulation GATA6 in hCMPCs reduces proliferation. miR-10a mimics and a plasmid that overexpresses GATA6 were cotransfected into hCMPCs, and a reduced BrdU absorbance was not observed. *P<0.05, n = 6.

We used an online algorithm (Targetscan) to search for potential targets of miR-10a that might explain its function in hCMPCs. Among the candidates, GATA6, a member of the zinc-finger family that is involved in cardiac development, attracted our attention. We found that miR-10a mimics decrease the luciferase activity by 53.75% compared to mock. Then, we cotransfected miR-10a and the luciferase reporter that did not contain the predicted binding site of the GATA6 3′UTR and found that the luciferase activity was not altered (**Figure 3A**). This suggests that miR-10a directly and specifically binds to the 3′UTR of GATA6.

We also verified whether miR-10a affects the protein expression of GATA6. Western blot showed that miR-10a mimics suppresses the expression of GATA6 by 19.72%, while its inhibitor increases GATA6 protein expression by 38.43% compared with mock (**Figure 3B, C**). Our findings indicate that miR-10a interferes with the expression of GATA6 by specifically binding its 3′UTR.

### GATA6 Overexpression Rescues the miR-10a-mediated Effect on hCMPCs

We explored whether suppression of the GATA6 protein by miR-10a could account for miR-10a′s inhibitory effect on hCMPCs. We used siRNA against a specific region of GATA6 to transfect hCMPCs. The GATA6-depressed hCMPCs displayed a 40.96% reduction of BrdU incorporation rate compared with control. We then cotransfected miR-10a mimics and the GATA6 overexpression plasmid into hCMPCs; overexpression of GATA6 rescued the decreased proliferation caused by miR-10a mimics (**Figure 3D** **, Figure S5 in File S1**). These data suggest that GATA6 is responsible for the miR-10a-mediated depressive effect on the proliferation of hCMPCs.

### Expression Pattern of miR-10a in Mouse Cardiogenesis

**Figure 4:**
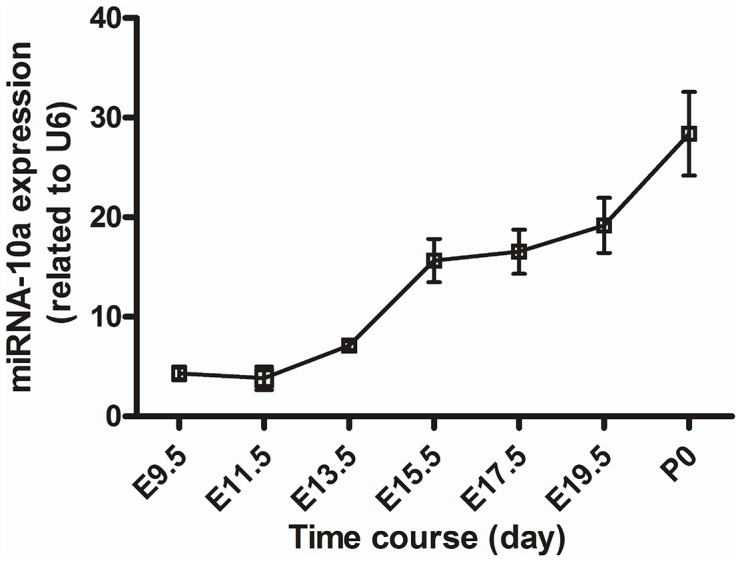
Expression of miR-10a is increased in cardiogenesis. Hearts of different stages of cardiogenesis were collected for miR-10a expression analysis. Quantification of miR-10a level was related to U6. *P<0.05, n = 6.

To determine the possible role of miR-10a in cardiogenesis, we examined the expression pattern of miR-10a in developing hearts from E9.5, E11.5, E13.5, E15.5, E17.5 and E19.5 mouse embryos and P0 mice. We found that miR-10a expression was relatively low in E9.5 and E11.5 hearts and gradually increased from E13.5, peaking at E15.5 through P0 (**Figure 4**). This evidence suggests that miR-10a is an important potential regulator in cardiac development.

## Discussion

miRNAs are emerging as important regulators of the proliferation and differentiation of embryonic stem cells and cardiac progenitor cells [19], [20]. miR-1 regulates cell lineage and promotes cardiac muscle progenitor differentiation in ES cells [20]. miR-1 and miR-499 reduce hCMPC proliferation and facilitates their differentiation into cardiomyocytes [21], [22]. Our previous study also indicated that miR-204 is required for hCMPC differentiation into cardiomyocytes [16]. In the present study, we provide solid evidence that miR-10a reduces the proliferation of hCMPCs by blocking G1/S transition and decreasing DNA synthesis, which leads to cell cycle depression.

GATA6 plays essential roles in modulating cardiac cells in cardiogenesis. In Xenopus, it was suggested that GATA6 maintains cardiomyocyte progenitor status and its down-regulation is required for myocardium differentiation [23]. GATA4 and GATA6 are functionally redundant in mice, and a threshold of expression of GATA4 and GATA6 is required for normal heart development [24], [25]. GATA4/6 compound heterozygotes display failed separation of the great vessels [25], indicating a defect in OFT extension, which is mainly attributed to the proliferation of progenitor cells from the second heart field [7]. The mutants also manifest a thin-walled myocardium and VSDs that are due to decreased proliferation of cardiac cells [25]. Transcription factors such as Nkx-2.5 have been suggested to be critical for GATA6 activation [26]. By the use of an online algorithm (Targetscan), we found that GATA6 is among the potential candidates targets of miR-10a. Our experimental study confirmed that miR-10a down-regulates the expression of GATA6 by directly binding to its 3′UTR and GATA6 overexpression rescues the miR-10a-mediated depressive effect on the hCMPCs proliferation. Thus, miR-10a could regulate the proliferation of hCMPCs by targeting GATA6. Other experiments are needed to determine how GATA6 is regulated by miR-10a in cardiogenesis.

miR-10a has been reported to regulate cell behaviors in different cell types. Several targets were identified and proved to mediate the regulation function of miR-10a [11]–[14]. Howerver, how miR-10a influence hCMPC cell processes has never been explored. In this work, we revealed the regulation role of miR-10a in hCMPC and confirmed GATA6 as the target. We also examined targets of miR-10a which has been reported to be involved in cardiogenesis and cell proliferation, including HDAC4 and HOXA1. No significant changes were observed in hCMPC transefected with miR-10a mimics (data not shown).

According to a previous study, miR-10a is enriched in human embryos, which is indicative of a role in embryonic development [27]. We have identified a group of differentially expressed miRNAs by microarray screening between human Tetralogy of Fallot (TOF) patients and healthy individuals in our previous work. miR-10a was down-regulated in individuals with TOF (∼2.5 fold), suggesting the potential role of miR-10a in cardiogenesis [28]. We showed that the expression of miR-10a increases at E13.5 and peaks at E15.5 through P0. Despite the lack of *in vivo* evidence, miR-10a is still an important potential regulator in cardiac development. Our evidence did not support that miR-10a influences the progression of cardiomyocyte differentiation from hCMPCs. This finding may be partly due to the fact that miR-10a does not significantly upregulate CKis, such as P21, which increases during myogenic induction [27]. Cardiomyocytes are the most abundant cells in the heart, and their abnormalities account for the majority of cardiac defects. Therefore, cardiomyocyte differentiation is examined in the present study. Further experiments are needed to determine whether miR-10a influences hCMPC differentiation into smooth muscle cells and endothelium.

In summary, our findings show that miR-10a reduces proliferation of hCMPC by suppressing GATA6. This study identifies a new regulator of hCMPC cell behavior and demonstrates a novel regulatory mechanism for the expression of GATA6. Further *in vivo* studies may determine the pathologic phenotypes of miR-10a in cardiogenesis.

## Supporting Information

File S1 Figure S1, Influence of miR-10a mimics or inhibitor on hCMPC viability. Figure S2, miR-10a mimics or inhibitor changes expression level of miR-10a. Figure S3, miR-10a decreases the proliferation of hCMPCs. Figure S4, miR-10a decreases hCMPCs proliferation without affects cell apoptosis. Figure S5. GATA6 rescues the proliferation phenotype of miR-10a in hCMPC.
(PDF)
