# Supplementary material for: miR-10a Regulates Proliferation of Human Cardiomyocyte Progenitor Cells by Targeting GATA6
Source: PLoS One. 2014 Jul 28;9(7):e103097. doi: 10.1371/journal.pone.0103097 (PMC4113387; doi:10.1371/journal.pone.0103097)
Supplement: File S1 — Figure S1, Influence of miR-10a mimics or inhibitor on hCMPC viability. Figure S2, miR-10a mimics or inhibitor changes expression level of miR-10a. Figure S3, miR-10a decreases the proliferation of hCMPCs. Figure S4, miR-10a decreases hCMPCs proliferation without affects cell apoptosis. Figure S5. GATA6 rescues the proliferation phenotype of miR-10a in hCMPC. (PDF) [file pone.0103097.s001.pdf]

Supplementary Figure Legends

Figure S1. Influence of miR-10a mimics or inhibitor on hCMPC viability. Different concentration of oligonucleatides were transfected into hCMPC, which then were used for CCK-8 test. *P<0.05, n=6.

Figure S2. miR-10a mimics or inhibitor changes expression level of miR-10a. hCMPC were transfected with 50nM miR-10a mimics or inhibitor, and proceeded to miR-10a expression detection by RT/PCR. *P<0.05, n=5.

Figure S3. miR-10a decreases the proliferation of hCMPCs. A. Representative image of hCMPCs transfected with mock, miR-10a mimics or inhibitor stained with crystal violet. to count cell numbers (×200). B. Quantify of the cell counting experiment showed miR-10a mimics decreased the hCMPCs proliferation. *P<0.05, n=3.

Figure S4. miR-10a decreases hCMPCs proliferation without affects cell apoptosis. The protein expression of caspase 3 was detected in the hCMPCs which were transfected with miR-10a mimics and an unrelated-oligo. n=3.

Figure S5. GATA6 rescues the proliferation phenotype of miR-10a in hCMPC. Representative images for EdU incorporation are showed (×200).

Figure S1

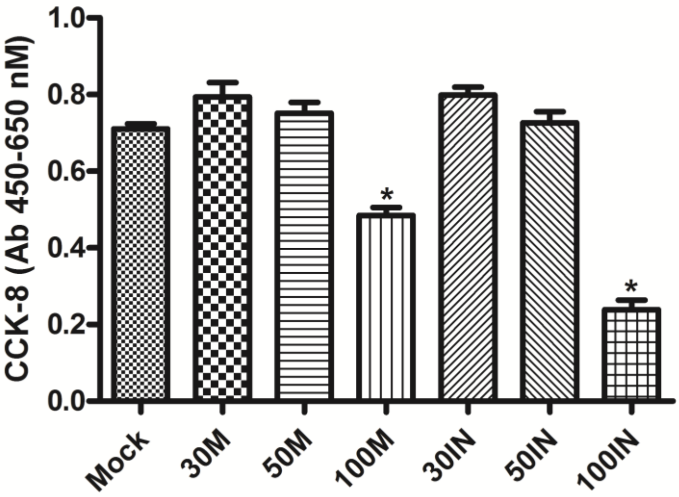

CCK-8 (Ab 450-650 nM)
1.0
0.8
0.6
0.4
0.2
0.0
*
*
Mock
30M
50M
100M
30IN
50IN
100IN

Figure S2

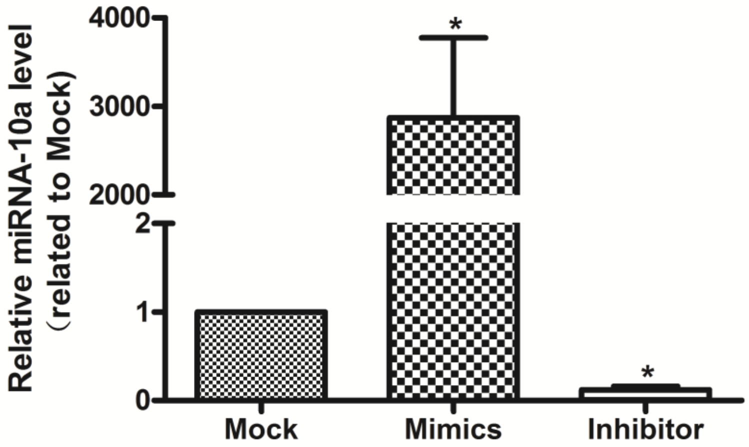

Relative miRNA-10a level
（related to Mock)
4000
3000
2000
2
1
0
*
*
Mock
Mimics
Inhibitor

Figure S3

A

Mock Mimics Inhibitor

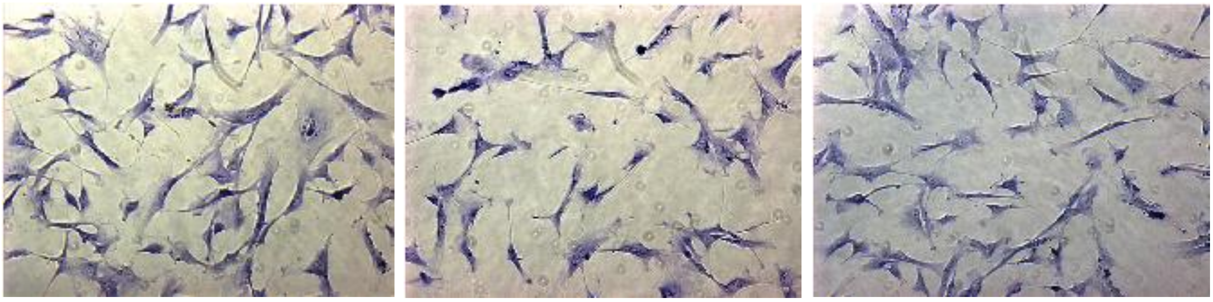

B

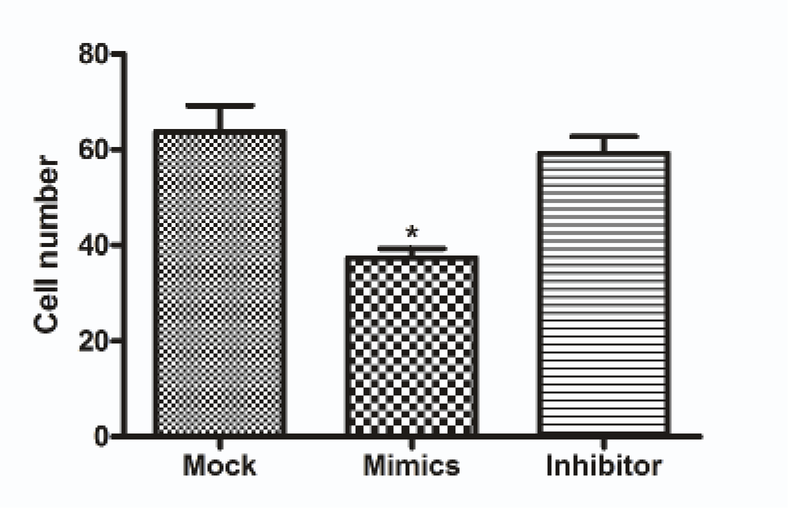

Figure S4

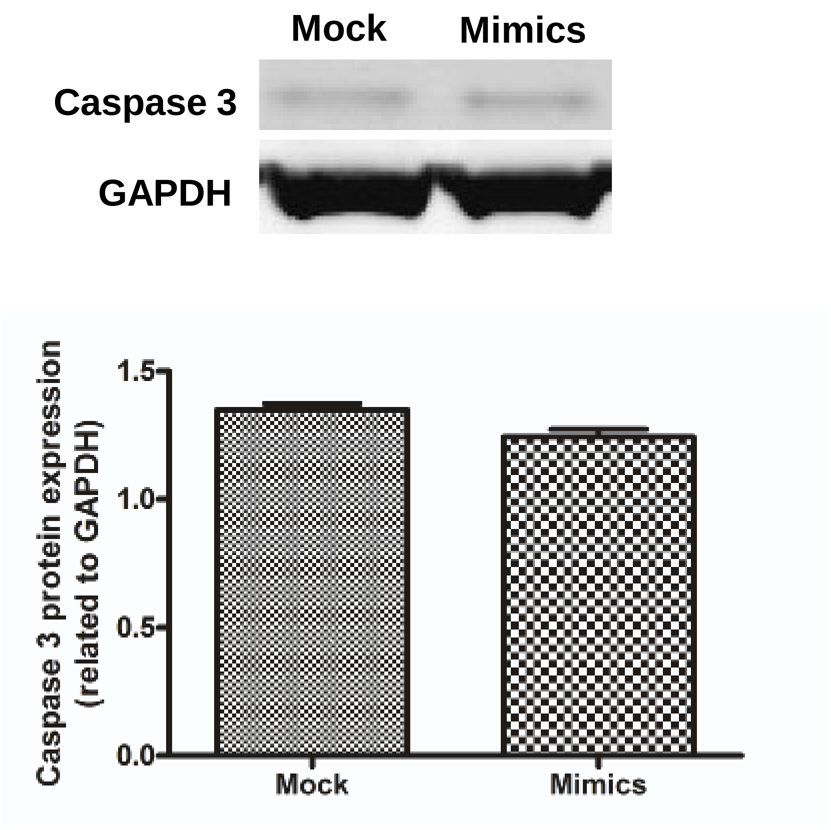

Mock
Mimics
Caspase 3
GAPDH
Caspase 3 protein expression (related to GAPDH)
1.5
1.0
0.5
0.0
Mock
Mimics

Figure S5

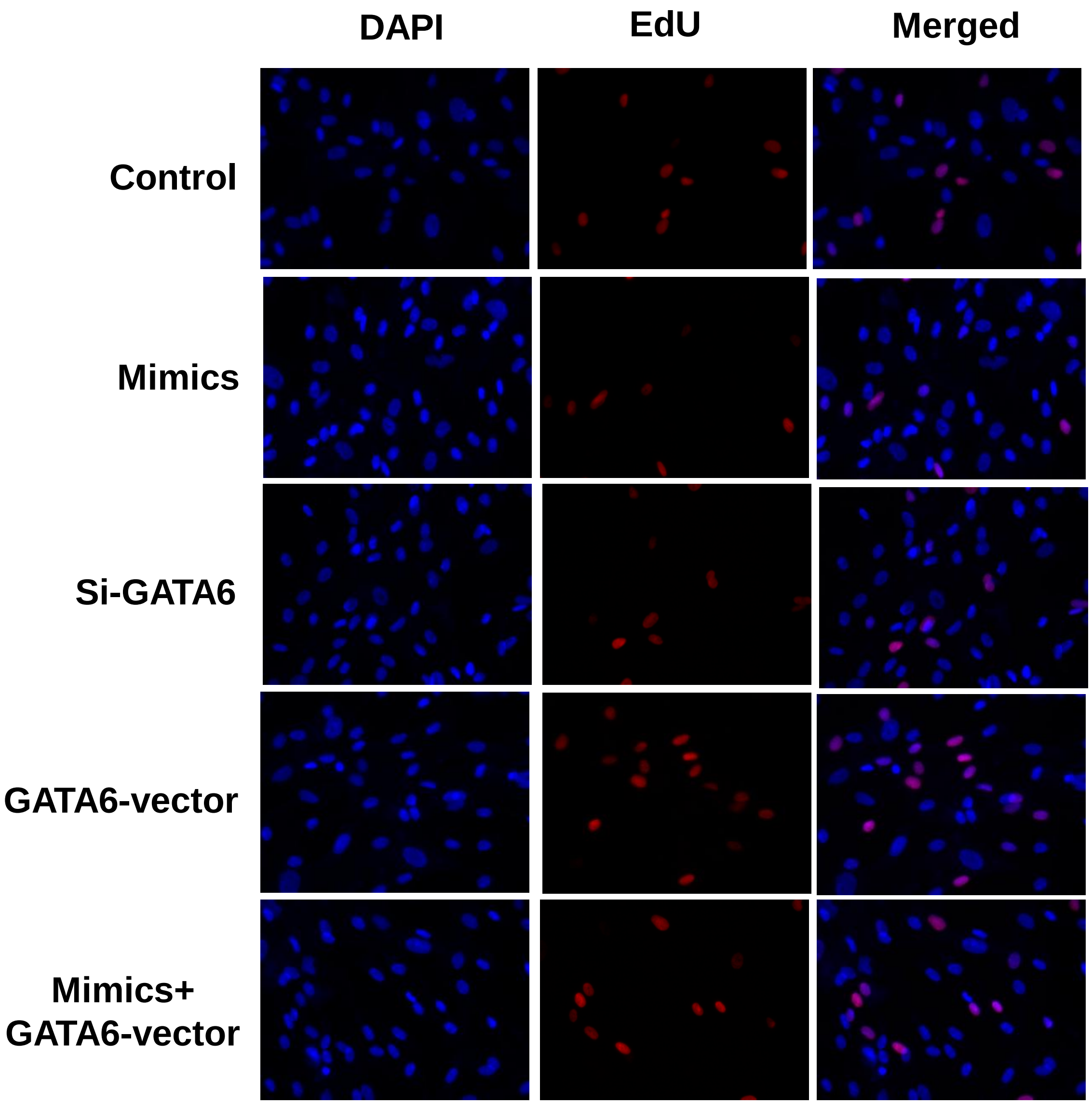

DAPI
EdU
Merged
Control
Mimics
Si-GATA6
GATA6-vector
Mimics+
GATA6-vector
